# Hemophagocytic Lymphohistiocytosis Complicating Systemic Sarcoidosis

**DOI:** 10.7759/cureus.2838

**Published:** 2018-06-19

**Authors:** Omar Abughanimeh, Ayman Qasrawi, Mouhanna Abu Ghanimeh

**Affiliations:** 1 Department of Internal Medicine, University of Missouri Kansas City School of Medicine, Kansas City, USA; 2 Department of Internal Medicine/Gastroenterology, Henry Ford Health System, Detroit, USA

**Keywords:** hlh, hemophagocytic lymphohistiocytosis, sarcoidosis, macrophage activation syndrome

## Abstract

Hemophagocytic lymphohistiocytosis (HLH) is a rare, life-threatening hyperinflammatory syndrome characterized by histiocyte proliferation and hemophagocytosis. Primary HLH is caused by genetic defects, whereas secondary HLH occurs in the setting of underlying diseases, such as infections, malignancies, or rheumatic diseases. Rheumatic diseases, such as systemic juvenile arthritis or systemic lupus erythematosus, have been associated with HLH. However, the association between sarcoidosis and HLH has been rarely reported in the literature. Herein, we report a case of a 36-year-old female who was recently diagnosed with sarcoidosis, and she developed fatal HLH that was not responsive to high-dose steroids.

## Introduction

Hemophagocytic lymphohistiocytosis (HLH) is a life-threatening syndrome, which results from histiocyte proliferation and extensive hemophagocytosis as a result of a hyperinflammatory response [1]. HLH can be classified as primary HLH, which is caused by genetic defects, or secondary HLH, which is caused by infections, malignancies, or rheumatic diseases [2]. Both primary and secondary HLH exhibit the same presentation of fever, hepatosplenomegaly, and pancytopenia [1-2]. The diagnosis and treatment of most HLH cases are performed by a protocol released by the Histiocyte Society called HLH-2004 (previously HLH-94) [3]. Early diagnosis is crucial for the promotion of early treatment; HLH is a disease that becomes rapidly fatal with mortality rates ranging between 8% and 22% [4]. Macrophage activation syndrome (MAS) is a term used to describe HLH that results from rheumatic or systemic diseases [3]. MAS is a rare disease, and it is typically associated with systemic juvenile arthritis [1]. Recently, MAS has been increasingly reported with other systemic diseases, such as systemic lupus erythematosus (SLE) and Still’s disease [1]. Sarcoidosis is a chronic systemic granulomatous disease that has different presentations [2]. The association between HLH and sarcoidosis is rare and not well reported.

## Case presentation

**Figure 1:**
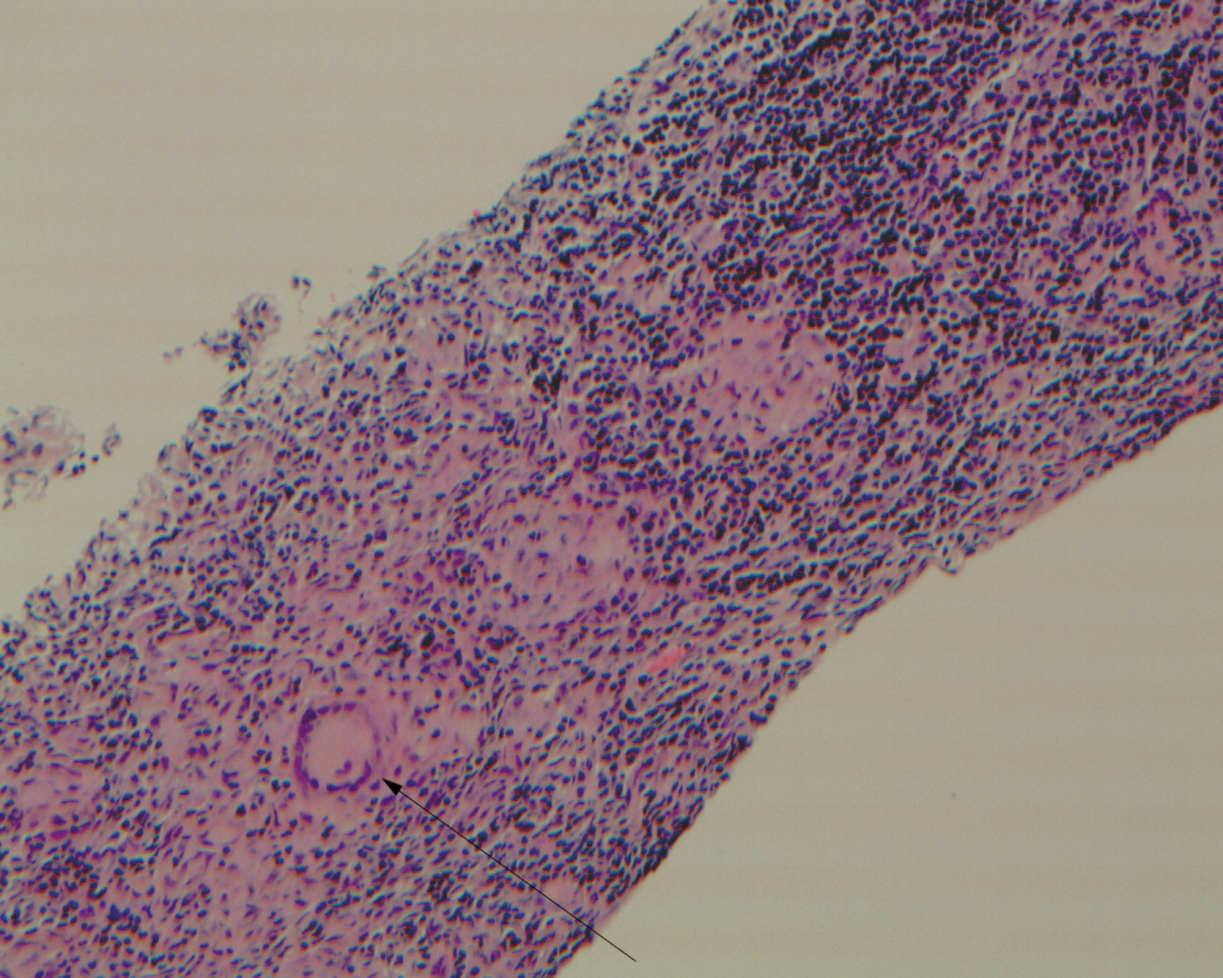
Lymph node biopsy showing noncaseating granulomas.

A 36-year-old female presented with fever, weight loss, enlarged right axillary lymph node, and bilateral pulmonary infiltrates. The axillary lymph node biopsy (Figure 1) revealed noncaseating granulomas typical for sarcoidosis. The patient was started on steroids and showed only mild improvement in symptoms. 

Over the next few months, she exhibited gradually increasing leukopenia and thrombocytopenia [white blood cell (WBC) count 1.5-4 × 109/l and platelet count 45-100 × 109/l]. A bone marrow biopsy was performed, and it confirmed the presence of noncaseating sarcoid granulomas. The patient was maintained on steroids, but no improvement in cytopenia was noted.

**Figure 2:**
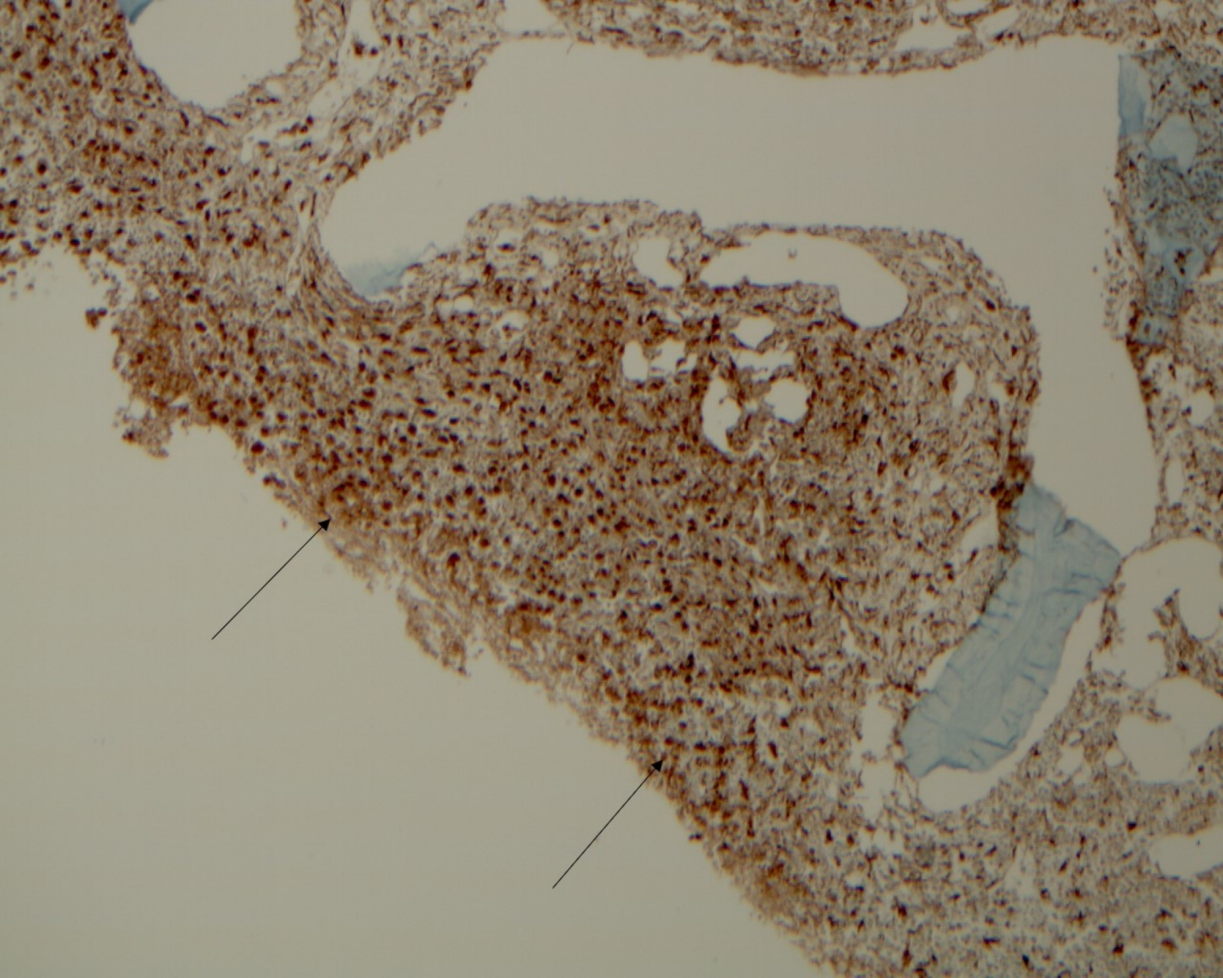
A bone marrow biopsy showing diffuse positive CD68 indicating histiocytes.

One year after diagnosis, she presented again with fever, fatigue, and hepatosplenomegaly. Laboratory workup revealed the following: WBC count 0.1 × 109/l, hemoglobin 5.5 g/dl, platelet count 9 × 109/l, total bilirubin 1.4 g/dl, aspartate aminotransferase (AST) 151 IU/l, alanine aminotransferase (ALT) 125 IU/l, lactate dehydrogenase (LDH) 317 U/l, alkaline phosphatase (ALP) 423 IU/l, and ferritin 67,590 ng/ml. The patient was treated with antibiotics but was persistently febrile. Blood cultures and viral and fungal studies were negative. Given worsening pancytopenia, very high ferritin and liver dysfunction, MAS was suspected. The fasting triglyceride level was 292 mg/dl, and the D-dimer level was 1001 ng/dl. However, the fibrinogen levels were normal. The sCD25 level was 10,440 pg/ml (ref < 1000 pg/ml) and the natural killer (NK)-cell activity was 4 LU30 (normal 7-125 LU30). A bone marrow biopsy (Figure 2) demonstrated hypocellular marrow with normal hematopoiesis replaced by proliferation of histiocytes and vague granulomas. Grocott-Gomori's methenamine silver (GMS) stain and acid-fast bacilli stain (AFB), CD1a, S100, and CD23 were negative. 

Magnetic resonance imaging (MRI) of the brain revealed diffuse pachymeningeal enhancement. Despite the absence of hemophagocytosis, she met the remainder of the criteria for HLH. The patient did not respond to a trial of intravenous immunoglobulin (IVIG) and 1 g/d methylprednisolone. She developed encephalopathy and status epilepticus and was transferred to another facility for escalated care. She died shortly after the transfer.

## Discussion

Hemophagocytic lymphohistiocytosis is a hyperinflammatory syndrome. It was first described in 1939 and is characterized by histiocyte proliferation and hemophagocytosis, which presents as cytopenia, fever, and hepatosplenomegaly [1-2, 5]. HLH can be classified according to the cause as primary HLH, which is due to genetic mutations, or secondary HLH, which is due to infections, autoimmune diseases, or malignancies [1]. Primary HLH is typically a pediatric disorder, and most cases present in the first two years of life. The disease has an incidence of 1/50,000 live births and exhibits an autosomal recessive inheritance pattern [1-2]. There are five known types of primary HLH known as familial hemophagocytic lymphohistiocytosis (FHL), which are caused by mutations at specific gene loci. FHL-1 is caused by a mutation in the *HPLH1* gene; FHL-2 is caused by a mutation in the *PRF-1* gene that codes for perforin protein; FHL-3 is associated with a mutation in the *UNC13D* gene that codes for Munc13–4 protein; FHL-4 is associated with mutations in *STX11* gene that codes for syntaxin 11 protein; and FHL-5 is caused by mutations in the *STXBP2* (*UNC18B*) gene that codes for MUNC18–2 protein [4]. Moreover, primary HLH can be associated with other mutations that lead to primary immunodeficiency syndromes, such as Chediak-Higashi syndrome, X-linked lymphoproliferative syndromes, and others [6]. On the other hand, secondary HLH can develop at any age but is typically a disease that manifests in adults. This condition occurs due to strong immune system activation due to infections (especially viral infections), rheumatic diseases, or malignancies, especially with lymphoma [1-2]. MAS is a relatively new term that is used to describe secondary HLH triggered by rheumatic diseases [7-8]. It was first suggested by Hadchouel et al. in 1985 who described a syndrome characterized by hematologic, hepatic, and neurologic abnormalities in seven children with juvenile rheumatoid arthritis [9]. Given that MAS is actually a secondary form of HLH, Athreya suggested the use of the term rheumatic disease-associated hemophagocytic syndrome (RAHS) to avoid confusion [10]. 

Rheumatic disease-associated hemophagocytic syndrome is not a common disease. Fukaya et al. [11] evaluated 1014 patients with systemic autoimmune diseases and identified 30 cases of HLH with a prevalence of 3%. Most of these cases were patients with SLE; none of the cases in this study were related to sarcoidosis. Atteritano et al. [1] performed a systemic review on 421 cases of rheumatic diseases associated with HLH. In this study, systemic juvenile arthritis was the most common disease associated with HLH (219 cases) followed by SLE (94 patients). Sarcoidosis was reported in only five patients. Among these five patients, two had documented active infection, including one with miliary tuberculosis and the other with histoplasmosis. Dhote et al. [12] reviewed 26 patients with systemic diseases and HLH. Among these patients, one had pulmonary sarcoidosis and developed HLH without any triggering factor. Table 1 summarizes the HLH cases associated with sarcoidosis reported in the English literature.

**Table 1:** Reported HLH cases associated with sarcoidosis in the English literature.

Reference	Age (Years)/Gender	Triggering factor	Treatment	Outcome
Bártholo et al. [2]	56/male	No	Methylprednisolone	Died
Phillips et al. [8]	69/male	Disseminated histoplasmosis	Corticosteroids, etoposide and cyclosporine. He was also given itraconazole	Cured
Dhote et al. [12]	73/female	No	Not known	Unknown
Balduini et al. [13]	23/female	No	Intravenous gamma globulin and prednisone	Cured
Lam et al. [14]	42/male	Miliary tuberculosis	Died before starting treatment	Died
Okabe et al. [15]	32/male	Epstein-Barr virus (EBV) infection	Methylprednisolone and intravenous immunoglobulin (IVIG)	Died
Okabe et al. [15]	68/female	EBV infection	Infliximab and cyclosporine A	Died
Abughanimeh et al. (this case)	36/female	No	Methylprednisolone and IVIG	Died

The pathogenesis of HLH is poorly understood. Studies suggested that dysregulation of inflammatory and immune systems can cause peripheral T-cell expansion and reduce NK cell activity, which contributes to the condition [1]. This dysregulation can also cause increased cytokine release from T-cells and macrophages, such as interleukin (IL)-1, IL-6, tumor necrosis factor-alpha (TNF-α), interferon gamma (IFN-γ), and soluble IL-2 receptor (sIL-2R). Upon this inflammatory response, proliferation and activation of antigen-presenting cells, including macrophages, histiocytes, and CD8+ T cells, promote the phagocytosis of other cells [2].

The HLH typically presents with fever, bicytopenia/pancytopenia, and hepatosplenomegaly. It occasionally presents with neurological symptoms, seizures, skin rash, jaundice, or lymphadenopathy [1, 6-7]. Laboratory workup in HLH is typically significant for high ferritin, hypertriglyceridemia, liver dysfunction, and coagulopathy with hypofibrinogenemia [1]. In the study described above by Fukaya et al. [11], fever was present in 87% of the patients, and 30% exhibited neuropsychiatric symptoms, such as seizures, coma, and mood disorders. Leukopenia and thrombocytopenia were noted in 87% of the cases. Interestingly, the D-dimer levels were elevated in 96% of the patients.

**Figure 3:**
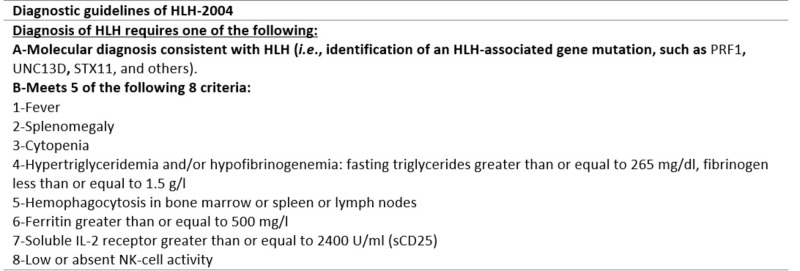
Diagnostic guidelines of HLH-2004.

The Histiocyte Society established the HLH-94 protocol to diagnose HLH. This protocol included five criteria (fever, splenomegaly, bicytopenia, hypertriglyceridemia and/or hypofibrinogenemia, and hemophagocytosis). This protocol was updated in HLH-2004 with the addition of three more criteria [3]. Figure 3 contains the most recent criteria to diagnose HLH.

Hemophagocytosis in bone marrow or other lymphoid tissues, such as liver, spleen or lymph nodes, is helpful for the diagnosis and identification of the secondary causes. However, it is not necessary to make a diagnosis as the biopsy does not demonstrate hemophagocytosis in approximately 20% of the cases [2, 6].

The HLH-2004 criteria have some limitations. First, the criteria were designed for pediatric populations rather than adults [6]. Moreover, elevated ferritin, splenomegaly, and pancytopenia can be observed in diseases other than HLH [2, 7]. Finally, testing for sCD25 and NK cell activity is expensive and not available at all centers. Efforts have been made to improve the accuracy of the current criteria. Fardet et al. [16] performed a retrospective cohort review for 312 patients with secondary HLH to develop a diagnostic score called “HScore.” This score depends on three clinical data (underlying immunosuppression, fever, and organomegaly including hepatomegaly and splenomegaly), five biological factors (ferritin, triglyceride, serum glutamic oxaloacetic transaminase—SGOT, cytopenia, and fibrinogen), and one cytologic factor (hemophagocytosis on bone marrow aspirate). This score was specifically designed for secondary HLH and may be worthy of validation in larger studies. It is available online: http://saintantoine.aphp.fr/score/

The Histiocyte Society developed treatment guidelines in 1994 known as the HLH-94 protocol. In this protocol, the treatment consisted of eight weeks of induction therapy of dexamethasone, etoposide, and intrathecal methotrexate for those with central nervous system involvement. Once the eight weeks are completed, patients are either weaned off therapy or transitioned to continuation therapy with cyclosporine A to prepare for hematopoietic cell transplantation [3-4,17]. This protocol was updated in 2004 to become the HLH-2004 protocol. This protocol had two major modifications. The first modification is the movement of cyclosporine to the beginning of the treatment, and the second modification is the addition of hydrocortisone to intrathecal methotrexate [17]. After the induction of therapy, cure might be achieved by hematopoietic cell transplantation [7]. Of note, HLH-2004 was designed for patients younger than 18 years old [6, 8]. Clinically, it is applied for primary HLH in patients less than 18 years old or patients with any severe form of HLH [4].

In general, HLH rapidly becomes a fatal disease, which makes early recognition crucial. Mortality ranges between 8% and 22% [1]. Without treatment, HLH has a median survival of two months [3]. Dohte et al. [12] studied 26 cases of HLH and concluded that absence of lymphadenopathy, thrombocytopenia, immunosuppressive therapies at the time of HLH diagnosis was associated with poor prognosis and increased mortality. Other studies have demonstrated that age greater than 50 years and C-reactive protein (CRP) level <50 mg/l are associated with increased mortality [11].

Our case was presented in the American Thoracic Society (ATS) 2017 International Conference (Abstract: Qasrawi A, Abu Omar M, Abu Ghanimeh M, et al. Fatal Macrophage Activation Syndrome Complicating Systemic Sarcoidosis. Am J Respir Crit Care Med. 2017; 195: Abstract issue; A3360) (https://www.atsjournals.org/doi/abs/10.1164/ajrccm-conference.2017.195.1_MeetingAbstracts.A3360).

## Conclusions

The association between sarcoidosis and HLH/MAS is extremely rare. Our patient had bone marrow involvement based on her sarcoidosis. However, worsening counts, high ferritin, and new onset liver dysfunction despite treatment with high-dose steroids and IVIG suggested possible HLH/MAS. Currently, no guidelines are available to guide HLH treatment in adults as the HLH-2004 trial was performed in a pediatric population. Further studies and trials should be performed on HLH cases in adults, which may aid in improving the high mortality of this disease.
